# Influence of *Toxoplasma gondii* Infection on Male Fertility: A Pilot Study on Immunocompetent Human Volunteers

**Published:** 2015

**Authors:** Horaţiu Alexandru COLOSI, Babak JALALI-ZADEH, Ioana Alina COLOSI, Laura Mihaela SIMON, Carmen Anca COSTACHE

**Affiliations:** 1 *Dept. of Medical Informatics and Biostatistics, “Iuliu Haţieganu” University of Medicine and Pharmacy, Cluj-Napoca, Romania*; 2 *Dept. of Urology, Municipal Hospital, Dej, Romania*; 3 *Dept. of Microbiology, “Iuliu Haţieganu” University of Medicine and Pharmacy, Cluj-Napoca, Romania*

**Keywords:** *latent toxoplasmosis*, *TOX-IgG*, *spermiogram*, *FSH*, *testosterone*

## Abstract

***Background:*** This study was conducted to investigate the influence of *Toxoplasma gondii* infection on spermatic and hormonal parameters in a pilot sample of immunocompetent human male subjects.

***Methods:*** This cross sectional, observational pilot study on 60 immunocompetent human male subjects aged between 18 and 60 yr old was conducted between 2012 -2013. Blind evaluation of serological markers of past *T. gondii* infection (TOX-IgG, TOX-IgM) was performed, along with individual spermiograms and determinations of follicle-stimulating hormone (FSH) and testosterone serum levels.

***Results:*** The overall prevalence of past *T. gondii *infection in the investigated immunocompetent male subjects was 25%. No statistically significant influence of *T. gondii *infection on sperm characteristics (ejaculate quantity, sperm count, motility, morphology) and serum levels of FSH or testosterone were found. Among possible predictors of a modified spermiogram studied by multiple logistic regression along with the *T. gondii *infection (age, smoking, alcohol consumption, fertility influencing malformations, infections, trauma or medication), only the presence of varicocele in the medical history of the studied subjects was found to significantly participate in the prediction of a modified spermiogram (P=0.0154). A necessary sample size of 994 subjects was computed in order to achieve a test power of 0.8 (80%) to discriminate an effect size of 8.89% estimated by our pilot study.

***Conclusions:*** Although our investigation did not demonstrate an influence of latent *T. gondii* infection on spermatic and hormonal parameters of immunocompetent male humans, the absence of such an influence cannot be affirmed, due to the limited sample size of our pilot study.

## Introduction

The causes of male infertility are poorly defined (1). Infertility affects about 7% of all men and among them, cases with unknown etiologies (idiopathic cases) of male infertility have been reported to have a prevalence of about 50% (2). *Toxoplasma gondii* is considered the most successful known parasite, currently infecting around 25% of the world’s human population (3). It is highly transmissible through the ingestion of tissue cysts found in undercooked, infected meat, or by swallowing of oocysts found in contaminated vegetables and water. Therefore, human infections by *T. gondii* are common, but, in most cases of immunocompetent humans, they are asymptomatic or minimally symptomatic and their effects may remain unnoticed (4), except for foetuses infected during pregnancy (5, 6).

The negative effects of *T. gondii *infection on the human reproductive system have been documented especially in female patients (7) and immunocompromised male patients (8, 9), but only few studies have been published in international literature regarding the effects of *T. gondii *infection on the reproductive system of immunocompetent males.

A systematic review of protozoan infections in the male genital tract stipulates the possibility of* T. gondii *producing testicular damage through secondary hypogonadism via hypothalamic-hypophyseal axis alterations (10). More recent researches performed an experimental *T. gondii *infection on a rat-model and found that toxoplasmosis can affect all spermatic parameters include motility, concentration and morphology (11). *T. gondii* infection enhanced testicular steroidogenesis in rats (12). Another study found a significantly decreased production of sperm cells but no sterility, at one month after experimental *T. gondii* infection of male mice (13). 

Few studies have been performed regarding the long-term effects of *T. gondii *infection on spermatic or hormonal parameters of human immunocompetent males. Our literature review identified only one such study, that was conducted on a Chinese population and concluded that *T. gondii* infection may affect men’s fertility and cause sterility (14).

Given the scarce amount of evidence found in literature, the aim of our research has been to investigate a possible influence of *T. gondii* infection on spermatic and hormonal markers of infertility in a pilot sample of immunocompetent human males and to determine the effect size of this potential influence, in order to compute the necessary sample size for future investigations.

Therefore, the objectives of our pilot study have been:

1. To compute the prevalence of *T. gondii* infection in the studied sample;
2. To compare the prevalence of abnormal spermiograms in *T. gondii *infected versus uninfected subjects;
3. To test the hypothesis of a potential influence of *T. gondii *infection on spermiogram characteristics of immunocompetent male subjects (ejaculate quantity, sperm pH, sperm count, motility after one hour, morphology) while accounting for the role of other factors (age, smoking, alcohol consumption, testicular trauma or malformations, fertility influencing infections or medication) in the etiology of modified spermiograms;
4. To test the hypothesis of a potential influence of *T. gondii *infection on serum levels of FSH and testosterone.

## Materials and Methods

***Study area***

Our observational pilot study has targeted immunocompetent male subjects aged between 18 and 60 years.

***Sample collection***

We studied serological markers of *T. gondii* infection and measured FSH and testosterone levels in a convenience sample of 60 immunocompetent male subjects who were also investigated by simple spermiogram between March 2012 and October 2013 in INTERMED Service Lab, Cluj-Napoca, Romania. 

We did not include subjects under 18 or above 60 years, subjects with congenital or acquired immune deficiency, subjects who refused to sign the informed consent of the study, subjects who did not offer both a sperm sample collected under correct conditions and a venous blood sample for serological investigation. After applying these exclusion criteria on 84 eligible subjects, our study sample included 60 subjects, which, according to criteria found in literature (15), constituted a reasonable sample size for a pilot study and its subsequent power analysis. 

Among these 60 subjects, 38 subjects (mean age ± SD = 31.79 ± 6.22 years; median=30.5 years; min.=20 years; max.=51 years) were permanent residents of Cluj County, Romania, who agreed to participate in the study after having requested urological advice on self-suspected fertility problems. 

The remaining 22 subjects (mean age ± SD = 25.41 ± 3.57 years; median=25 years; min.=19 years; max.=38 years) were male volunteers from four other European countries, temporary residents of Cluj County, Romania, who agreed to participate in the study after reading recruitment flyers distributed among students enrolled at the Medical University of Cluj-Napoca, Romania. The distribution of the 60 enrolled subjects by country of permanent residence has been as follows: Romania – 38 (63%), Sweden – 18 (30%), Germany – 2 (3%), France – 1 (2%), Norway – 1 (2%).

***Data collection***

All included subjects answered a questionnaire on their demographic, behavioral and health history. They were instructed to collect properly a sperm sample after five days of abstention. A venous blood sample has been collected for every subject, under sterile conditions. 

Sperm samples were examined and evaluated using WHO normality criteria of standard spermiograms.

Sperm samples and blood samples were labelled using a unique code that allowed for their blind evaluation. Blood samples were centrifuged and serum was analysed for the presence of TOX-IgG and TOX-IgM, using a standardized ELISA technique (commercial kit Platelia ^TM^, Bio-Rad, Marnes-la-Coquette, France), by an investigator unaware of subject identity and spermiogram results. From the same serum samples, follicle-stimulating hormone (FSH) and testosterone levels have been determined using an IMMULITE 2000 analyzer system (Siemens, Berlin, Germany).

***Statistical analysis***

Data description has been performed by computing frequencies and their 95% confidence intervals. Hypothesis testing for categorical variables has been performed by applying Fisher’s exact test. Normality has been evaluated for all quantitative variables using Q-Q plots and a Kolmogorov-Smirnov normality test. Hypothesis testing for quantitative variables has been performed by applying Student’s t-test for normally distributed variables and the Mann-Whitney test for non-normally distributed variables. The level of statistical significance has been set at α=0.05.

The role of other factors besides *T. gondii* infection in the etiology of modified spermiograms (age, smoking, alcohol consumption, fertility influencing malformations, infections, trauma or medication) has been investigated by multiple logistic regression. 

Study power analysis and necessary sample size computation have been performed using G*Power 3.1.9.

Data description and analysis have been performed using Microsoft Excel 2003, Epi Info 7 and R 2.15.1- software environment for statistical computing and graphics.

***Ethical approval***

The research protocol of this study has been evaluated and approved by the Ethics Committee of the “Iuliu Haţieganu” University of Medicine and Pharmacy, Cluj-Napoca, Romania. 

All eligible research subjects were asked to sign an informed consent form containing a presentation of the intended study, describing the blood and semen sampling methods and guaranteeing total confidentiality of personal data in the process of data analysis and results publication. Subjects who refused to sign the informed consent have not been included in the studied sample. 

## Results

Fifteen of the 60 subjects included in our pilot study presented serological evidence of past *T. gondii *infection (TOX-IgG positive). None of the investigated 60 subjects were TOX-IgM positive. 

The overall prevalence of past *T. gondii *infection in the investigated immunocompetent male subjects was thus estimated at 25% (95% CI 15.78-37.23%). This overall prevalence of past *T. gondii *infection was distributed as follows: 2 of the 22 volunteer students, representing 9.09% (95% CI 2.53-27.81%), and 13 of the 38 male urology patients with self-suspected subfertility, representing 34.21% (95% CI 21.21-50.11%), presented serological evidence of past *T. gondii *infection (TOX-IgG positive). The 25.12% difference in prevalence of past *T. gondii *infection among the two groups was statistically significant (*P*=0.028 – Fisher’s exact test).

In the overall sample, six of the fifteen subjects with a history of *T. gondii *infection (TOX IgG+), representing 40% (95% CI 19.82-64.25%), exhibited a modified spermiogram, compared to 14 of the 45 subjects without a history of *T. gondii *infection (TOX IgG-), representing 31.11% (95% CI 19.53-45.66%). The 8.89% difference in prevalence of modified spermiograms among subjects with a history of *T. gondii *infection compared to uninfected subjects did not reach statistical significance (*P*=0.37 – Fisher’s exact test) in our pilot study sample.

**Fig. 1:**
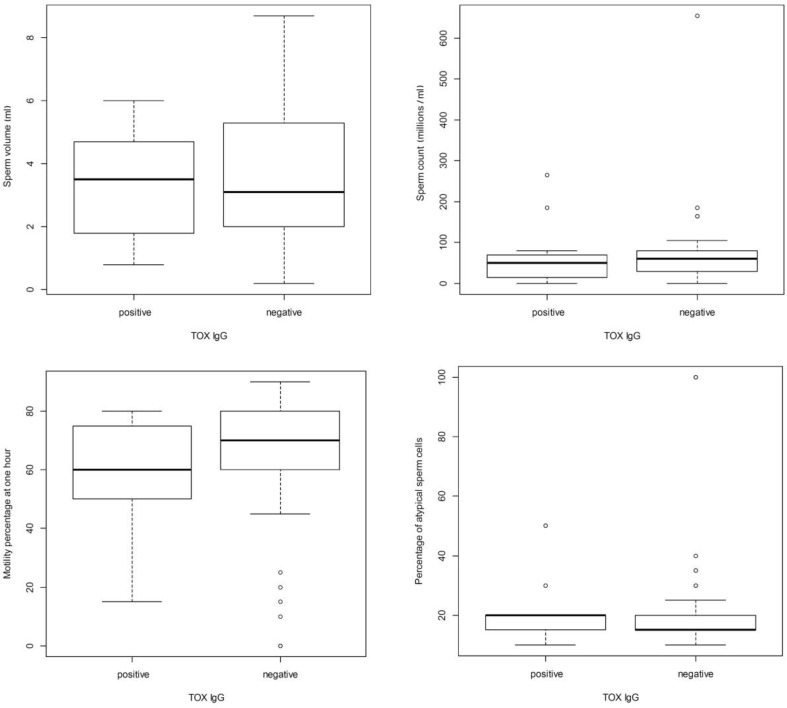
Quartile distributions of the investigated sperm characteristics in Toxoplasma gondii infected subjects compared to non-infected subjects. Circles represent outside values; whiskers represent the highest, respectively lowest datum still within 1.5 interquartile range from the first, respectively second quartile

No statistically significant influence (*P*>0.05 – Mann-Whitney test) of *T. gondii *infection on sperm characteristics (ejaculate quantity, sperm count, motility, morphology) has been found (Fig. 1).

Among possible predictors of a modified spermiogram studied by multiple logistic regression along with the *T. gondii *infection (age, smoking, alcohol consumption, fertility influencing malformations, infections, trauma or medication), only the presence of varicocele in the medical history of the studied subjects has been found to participate significantly in the prediction of a modified spermiogram (Table 1). 

**Table 1:** Multiple logistic regression analysis of other factors besides Toxoplasma gondii infection in the etiology of a modified spermiogram (dependent dichotomous variable)

**Factor**	**Odds Ratio**	**95% CI**	**Coefficient**	***P*** **-value**
Age	1.0470	0.9447 - 1.1604	0.0459	0.3813
Alcohol	0.8096	0.1894 - 3.4614	-0.2112	0.7757
Inguinal hernia	1.9991	0.3325 - 12.0173	0.6927	0.4491
Orchiepididimitis	0.8005	0.0552 - 11.6057	-0.2225	0.8704
Smoking	2.9336	0.6535 - 13.1695	1.0762	0.1601
TOX IgG+	1.3296	0.3236 - 5.4637	0.2849	0.6927
Testicular trauma	0.7545	0.0471 - 12.0903	-0.2817	0.8423
Varicocele	6.3447	1.4225 - 28.2987	1.8476	0.0154*
CONSTANT			-2.7910	0.0770

* significant at a level of 0.05

Factors such as radiation exposure, cryptorchidism, hormone or contraceptive treatments, and other fertility influencing medication have only been found episodically and have not been included in the logistic regression analysis.

No statistically significant influence of *T. gondii *infection on seric levels of follicle-stimulating hormone (FSH) or testosterone has been found (Table 2).

**Table 2:** FSH and testosterone serum levels in Toxoplasma gondii infected vs. non-infected subjects

**Hormone**	**Group**	**N**	**Median**			**Mean**	**SD**		***P*** **-value**	
FSH (mIU/ml)	TOX IgG+	15	4.55			5.25	2.60		0.97	
	TOX IgG-	45	4.45			5.28	3.55			
Testosterone (ng/dl)	TOX IgG+	15		344	399.07			185.18	0.62
	TOX IgG-	45		372	425.96			170.05	

Stratified analysis, performed separately on the 38 urologic patients from Cluj County, Romania, did not find significant differences in spermatic (*P*>0.05 – Mann-Whitney test) and hormonal parameters between *T. gondii* infected and uninfected subjects.

A necessary sample size of 994 subjects has been computed in order to achieve a test power of at least 0.8 (80%) to discriminate the effect size of 8.89 % estimated by our pilot study.

## Discussion

The negative effects of *T. gondii* infection on male fertility have been suggested in literature by several investigations of short-term effects induced by experimental *T. gondii* infection on the spermatic and hormonal parameters of rat and mice (11-13). 

Nevertheless, whether the negative effects found in animal models are permanent or reversible, and whether these effects and their underlying mechanisms apply to other mammalian hosts, remains to be determined (13).

Since there is a lack of published evidence regarding the long-term effects of chronic infection with *T. gondii*, on the fertility of immunocompetent human males, a pilot study was necessary in order to pave the way for more extensive clinical investigations regarding the possible long-term effects of *T. gondii* infection on spermatic and hormonal markers of infertility in immunocompetent human males. 

Subject to sample size limitations inherent to a pilot study, the first objective of our investigation has been met by estimating an overall prevalence of 25% for *T. gondii *infection in immunocompetent males permanently and temporarily residing in Cluj County, Romania. As expected, due to the sampling of both permanent and temporary residents of Cluj County, this overall prevalence was lower than the prevalence of *T. gondii *infection found by previous studies performed in the same geographic region (16-18). However, the prevalence of *T. gondii *infection in the 38 permanent residents of Cluj County, computed by our study at 34.21% (95% CI 21.21-50.11%), came much closer to the *T. gondii *infection prevalence of 44.9% found in Cluj County (17).

Our study showed a significant difference between the prevalence of *T. gondii *infection in the 38 urology patients permanently residing in Cluj County and the 9.09% (95% CI 2.53-27.81%) prevalence found in the 22 volunteer students permanently residing in other, mainly North-European countries. This difference in prevalence may be explained, in part, by the younger age of the 22 students (mean age ± SD = 25.41 ± 3.57 years) compared to the 38 urology patients permanently residing in Cluj County (mean age ± SD = 31.79 ± 6.22 years), thus yielding a shorter exposure time in which to acquire the parasite, and, in part, by a potentially different exposure pattern to *T. gondii* strains, due to their medical education and a possibly lower exposure to the parasite in their countries of origin before their temporary residence in Romania for the past 4-5 academic years. This significant difference in prevalence highlights the problem that self-suspected subfertility, as well as other characteristics of the two sampled populations (urological patients and students) might have confounded or biased our study. Consequently, we decided to perform a stratified analysis of spermatic and hormonal parameters in these two groups, which did not find significant differences in spermatic or hormonal parameters between *T. gondii* infected and uninfected subjects. 

Therefore, with an achieved post-hoc power of just over 0.1 (10%), our pilot study was not able to demonstrate an influence of *T. gondii* infection on spermatic and hormonal parameters of immunocompetent male humans. Nevertheless, by estimating a necessary sample size, our preliminary study paved the way for more extensive investigations that will ensure adequate statistical power.

Among the factors that were investigated in our study, the presence of varicocele in the medical history of a patient was the only one that contributed significantly to the prediction of an abnormal spermiogram. 

The hypothesis of a potential influence of *T. gondii* infection on serum levels of FSH and testosterone could also not be confirmed by our pilot study. 

Our results, found in humans, seem to contradict some results found in rat models, which suggested an enhanced testicular steroidogenesis induced by *T. gondii*, either by the presence of *T. gondii* cysts in the rat’s brain and an increased production of luteinizing hormone, or directly by the presence of *T. gondii* cysts in the testis (12). 

Another study, however, found that toxoplasmosis-related impairments on reproductive parameters of male rats included a decrease of serum testosterone levels and sperm counts in infected male rats compared to controls, but these impairments were only temporary and reversible (19), therefore not likely to persist in latent *T. gondii* infections.

Nevertheless, another study found higher testosterone levels in *T. gondii* infected men and suggested an inverse causation mechanism, according to which lower natural resistance to *T. gondii* infection may be due to pre-existing high testosterone levels (20). 

As also suggested by a recent systematic review (21), given this contradictory evidence and the limited number of studies that have investigated the influence of latent toxoplasmosis on male fertility, more extensive clinical studies, as well as studies to clarify the underlying mechanisms are needed in order to elucidate the effects of *T. gondii* infection on male fertility.

## Conclusions

Our investigation did not demonstrate an influence of *T. gondii* infection on spermatic and hormonal parameters of immunocompetent male humans, but the absence of such an influence cannot be affirmed, due to the limited sample size of our pilot study. Nevertheless, by estimating a necessary sample size, our preliminary study paves the way for more extensive clinical investigations, able to ensure adequate statistical power.
